# MitoTracker transfers from astrocytes to neurons independently of mitochondria

**DOI:** 10.1016/j.crmeth.2026.101338

**Published:** 2026-03-13

**Authors:** Katriona L. Hole, Rosalind Norkett, Emma Russell, Patrick Cottilli, Molly Strom, Jack H. Howden, Nicola J. Corbett, Janet Brownlees, Michael J. Devine

**Affiliations:** 1 Mitochondrial Neurobiology Laboratory, The Francis Crick Institute, 1 Midland Road, London NW1 1AT, UK; 2 Biological Research Facility, The Francis Crick Institute, 1 Midland Road, London NW1 1AT, UK; 3 Vector Core, Human Biology Facility, The Francis Crick Institute, 1 Midland Road, London NW1 1AT, UK; 4 MSD (UK) Limited, 120 Moorgate, London EC2M 6UR, UK; 5 Department of Clinical and Movement Neurosciences, UCL Queen Square Institute of Neurology, University College London, London WC1N 3BG, UK

**Keywords:** neuron, astrocyte, intercellular mitochondrial transfer, MitoTracker, mitochondria

## Abstract

The neuroprotective transfer of mitochondria from astrocytes to neurons has been primarily investigated by labeling astrocytic mitochondria with the dye MitoTracker. Here, we labeled astrocytic mitochondria with both a genetically encoded fluorophore (GFP) and MitoTracker dye and then imaged neurons immediately after co-culture with astrocytes or astrocyte-conditioned media (ACM). We report that MitoTracker transfers to neurons from both astrocytes and ACM, independently of mitochondrial transfer. Our observations provide an essential caveat to the use of this reagent and suggest that the investigation of astrocyte-neuron mitochondrial transfer, and other systems in which contact-independent transfer has been reported, requires the use of alternative labeling techniques.

## Highlights

• We labeled astrocyte mitochondria with GFP and MitoTracker before co-culture
• We did not detect mitochondrial transfer from astrocytes to neurons
• MitoTracker rapidly transferred from astrocytes and conditioned media to neurons
• MitoTracker transfer does not equate to mitochondrial transfer

## Motivation

The phenomenon of intercellular mitochondrial transfer (IMT) from astrocytes to neurons is primarily supported by experiments using the dye MitoTracker to label astrocytic mitochondria. It was recently shown in other cell types that MitoTracker dye can transfer from donor to acceptor cells independently of mitochondrial transfer. However, MitoTracker dye transfer in these cells was dependent on cell-cell contact, while astrocyte-to-neuron IMT is reportedly contact independent. It remained unclear whether MitoTracker could similarly transfer between these cell types, independently of IMT. We aimed to clarify this by dual labeling astrocytic mitochondria with genetically encoded GFP and the MitoTracker dye. This enables direct comparison of dye versus mitochondrial transfer to neurons.

## Graphical abstract

**Figure:**
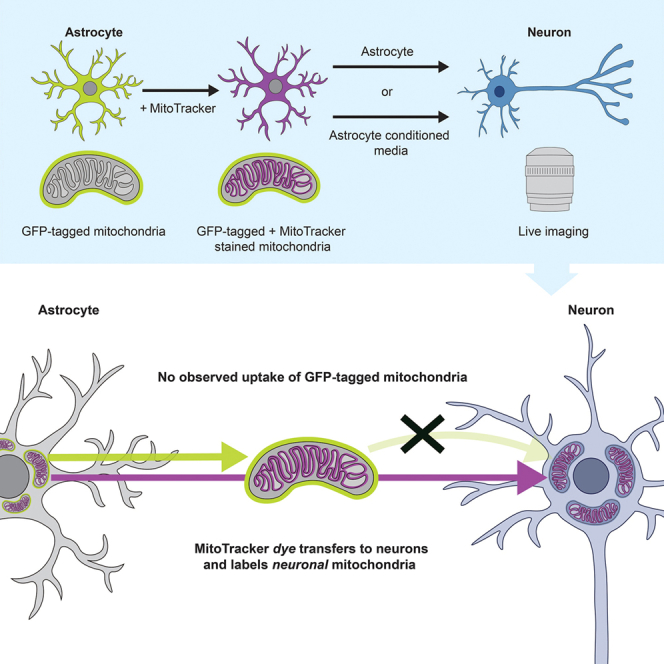

## Teaser

The mitochondrial dye MitoTracker is commonly used to investigate intercellular mitochondrial transfer (IMT), particularly between astrocytes and neurons. Hole et al. compare MitoTracker with a genetically encoded mitochondrial fluorophore and demonstrate that MitoTracker can transfer from astrocytes to neurons in the absence of mitochondrial transfer, without requiring cell contact.

## Introduction

Intercellular mitochondrial transfer (IMT) from astrocytes to neurons has been reported in 16 papers from 15 independent groups since the first report in 2016.^1^^,^^2^^,^^3^^,^^4^^,^^5^^,^^6^^,^^7^^,^^8^^,^^9^^,^^10^^,^^11^^,^^12^^,^^13^^,^^14^^,^^15^^,^^16^

From these reports, it has been concluded that (1) astrocytes can release functionally intact mitochondria, either within extracellular vesicles (EV-mitochondria) or unenveloped, and (2) neurons co-cultured with astrocytes or astrocyte-conditioned media (ACM) can take up astrocytic mitochondria. This transfer was shown to be upregulated following the application of mitochondrial stress to neurons, including oxygen/glucose deprivation,^2^^,^^10^^,^^11^^,^^15^ cisplatin,^1^ or rotenone.^9^ Furthermore, increased transfer correlates with improved neuronal viability.^1^^,^^2^^,^^9^^,^^10^^,^^11^^,^^15^ Notably, this neuroprotection can be observed *in vivo*, where transplantation of astrocytic mitochondria to the brains of mice can mitigate neuronal damage in models of ischemia.^2^^,^^12^^,^^15^

To study IMT, it is essential to selectively label mitochondria in the donor cell.^17^^,^^18^^,^^19^ For astrocyte-neuron transfer, almost all studies have employed the mitochondrial dye MitoTracker (mainly CMXRos but also Green/Deep Red) to label astrocytic mitochondria.^4^^,^^5^^,^^6^^,^^7^^,^^8^^,^^9^^,^^10^^,^^11^^,^^12^^,^^13^^,^^14^^,^^15^^,^^16^ However, a recent report showed that MitoTracker, which binds thiol-groups in mitochondria,^20^ can transfer between macrophages, B16 cells, HEK 293T cells, and immortalized bone marrow-derived cells independently of mitochondrial transfer.^21^ This dye transfer was found to be dependent on cell contact in the systems that were investigated, with no transfer observed in transwell co-cultures where donor and acceptor cells are physically separated. However, many MitoTracker-based studies have shown that astrocytic mitochondria can transfer through transwells and via ACM.^4^^,^^5^^,^^6^^,^^7^^,^^11^^,^^13^^,^^14^^,^^15^ Therefore, it is currently unclear to what extent reported astrocyte-neuron IMT observed with dyes is genuine, or an artifact of dye transfer. The aim of this study was to investigate the validity of MitoTracker dye as a robust reporter of IMT. We set out to clarify whether MitoTracker dye can transfer from astrocytes to neurons independently of mitochondria. By dual-labeling astrocytic mitochondria with genetically encoded GFP and MitoTracker dye, we show that MitoTracker transfers from astrocytes to neurons independently of genetically labeled mitochondria, without requiring cell contact.

## Results

**Figure 1:**
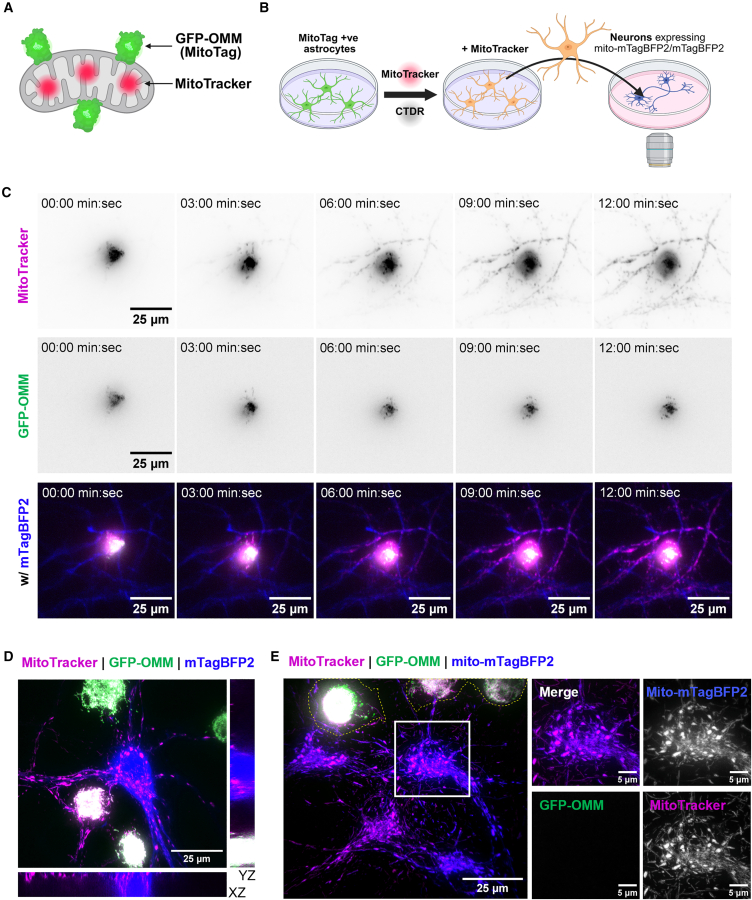
MitoTracker, but not mitochondria, transfers rapidly to neurons from astrocytes (A) Mitochondria are dual labeled with outer mitochondrial membrane targeted GFP (GFP-OMM) and the MitoTracker dye. (B) Schematic diagram outlining the protocol for live co-culture experiments. Astrocytes are additionally labeled with CTDR (CellTracker Deep red) and neurons/neuronal mitochondria are identified by mTagBFP2 and mito-mTagBFP2 expression, respectively. (C) Timelapse images of a dual-labeled astrocyte (central) immediately following co-culture with neurons. (*n* = 3 biological repeats, 4–5 positions of interest per repeat). (D) An orthogonal view of co-cultures following timelapse acquisition (>30 min). (E) Representative image of dual-labeled astrocytes co-cultured with mito-mTagBFP2 expressing neurons for >30 min. The yellow dashed line represents the astrocyte boundary. Insert highlights that MitoTracker but not GFP-OMM labels neuronal mitochondria. See also Video S1.

In order to genetically label mitochondria in the whole population of astrocytes, we generated primary cortical astrocyte cultures from MitoTag x GFAP*-cre* mice,^22^ where outer-mitochondrial membrane-targeted GFP (GFP-OMM) is expressed exclusively in astrocytes. Quantification confirmed that >97% of cells in astrocyte cultures were positive for GFP-OMM (Figures S1A and S1B). We also confirmed that GFP-OMM effectively labeled astrocytic mitochondria by direct comparison with matrix-targeted mito-DsRed2 introduced by lentiviral infection (Figure S1C). These astrocytes were then co-labeled with the mitochondrial dye MitoTracker and washed thoroughly to eliminate any residual extracellular dye (Figures 1A and 1B). Using these dual mitochondrially labeled astrocytes, we set out to compare the transfer of dye versus the transfer of mitochondria labeled with the genetically encoded tag.

We first compared MitoTracker and GFP-OMM transfer in neuron-astrocyte co-cultures because mitochondrial dye transfer was previously shown to be contact dependent.^21^ By imaging co-cultures immediately after application of astrocytes to primary cortical neurons expressing cytoplasmically targeted mTagBFP2, we observed that MitoTracker rapidly labeled mitochondria within adjacent neurons after a few minutes (Figure 1C and Video S1). However, GFP-OMM, which is confined to astrocytic mitochondria, did not transfer to neurons within the acquisition period of 30 min. Therefore, no IMT occurred over this brief time frame. Following acquisition of timelapses, higher magnification imaging confirmed that MitoTracker, but not GFP-OMM, was within neurons (Figure 1D). Furthermore, the transferred MitoTracker signal colocalized with neuronal mitochondria, as shown by co-culture with neurons expressing mitochondrial-matrix targeted mTagBFP2 (mito-mTagBFP2) (Figure 1E). The transfer of mitochondrial dye independently of mitochondria was also observed with the mitochondrial membrane potential-dependent dyes MitoTracker deep red and TMRM (Figures S1D and S1E). Additionally, MitoTracker Green, which labels mitochondria independently of mitochondrial membrane potential, transferred from astrocytes to neurons within the same time frame (Figure S1F). To confirm that mitochondrial transfer was not inhibited by the outer mitochondrial membrane targeting of GFP, we also co-cultured neurons with astrocytes lentivirally infected with mitochondrial matrix-targeted DsRed2 (mito-DsRed2). As with GFP-OMM, no mitochondrial transfer was detected with mito-DsRed2 (Figure S1G).

Video S1. MitoTracker labels adjacent neuronal mitochondria without mitochondrial transfer, related to Figure 1 Image acquisition immediately after astrocytes expressing GFP-OMM (green) and labeled with MitoTracker dye (magenta) are co-cultured with neurons expressing mito-mTagBFP2 (blue).

**Figure 2:**
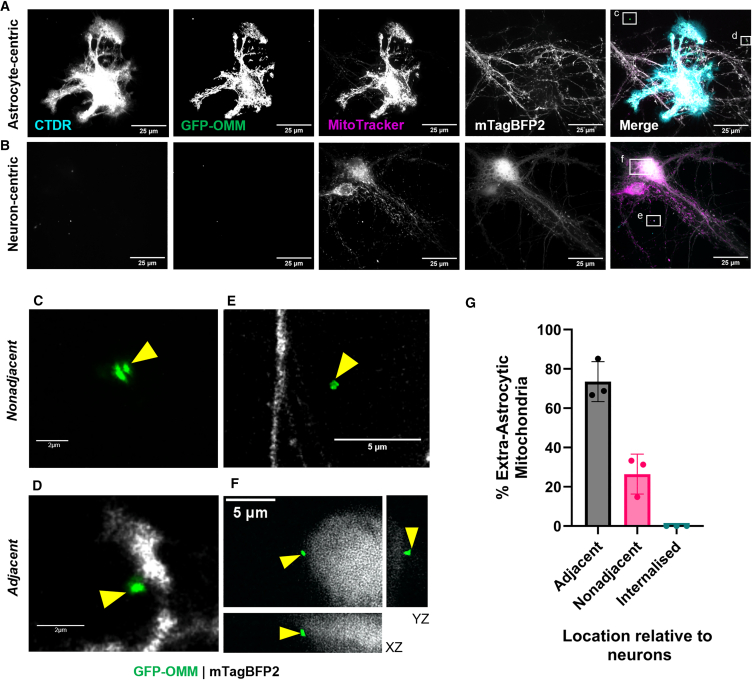
Longer astrocyte-neuron co-cultures show mitochondrial release but not internalization (A and B) Representative images after 48 h co-culture of neurons with astrocytes and immunolabeling against GFP. (C–F) Insets from (A and B) showing extracellular astrocytic mitochondria that are nonadjacent (C and E) or adjacent (D and F) to neurons. All images are maximum projections except (F) which shows an orthogonal view. Yellow arrows = astrocytic mitochondria. (G) Classification of extra-astrocytic mitochondria based on their location relative to neurons shown as a percentage of total extra-astrocytic mitochondria identified. *n* = 3 biological repeats, 30–54 mitochondria per repeat, 132 mitochondria in total. Data are presented as mean ± SD, with individual biological repeats shown.

We also compared GFP-OMM and MitoTracker transfer after co-culturing neurons and astrocytes for 48 h, a typical experimental duration used in previous reports.^8^^,^^15^ Theoretically, transferred GFP-OMM labeled mitochondria could be engulfed with autophagosomes and then fuse with acidic lysosomes for degradation—in which case the GFP fluorescence would be quenched rendering it undetectable. To ensure that all GFP-OMM-labeled mitochondria were detected, including those within acidified compartments, we amplified the GFP signal with anti-GFP immunofluorescence (Figure S2A). After 48 h, MitoTracker signal was still present in neurons, however, GFP-OMM-labeled mitochondria remained undetectable within neurons (Figures 2A and 2B). Instead, GFP-OMM-labeled mitochondria could be identified extracellularly to astrocytes (Figures 2A–2F). More than two-thirds of these extracellular astrocytic mitochondria were adjacent to neurons but none were transferred to neurons (Figures 2D, 2F, and 2G). Therefore, these results suggest that in contact co-cultures, mitochondrial dye transfers rapidly from astrocytes to neurons without concomitant transfer of mitochondria.

**Figure 3:**
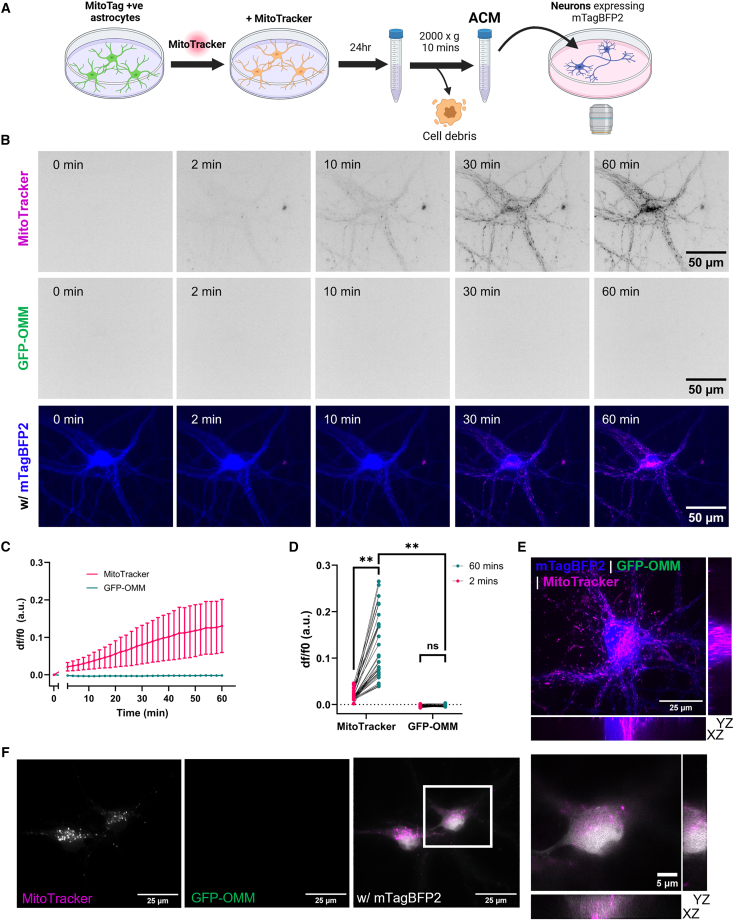
MitoTracker can transfer to neurons without direct cell-cell contact (A) Schematic diagram outlining the protocol for live ACM incubation experiments. (B) Timelapse microscopy of neurons incubated with ACM over 60 min (C) The mean somatic fluorescence intensity over time for neurons incubated with ACM, normalized to *t* = 0. The data are presented as mean ± SD. (D) Comparison of the somatic fluorescence intensity at 2 and 60 min for MitoTracker and GFP-OMM. The data are presented as measurements from individual cells, with statistics undertaken on biological repeats. Two-way repeated measures ANOVA with Uncorrected Fisher’s LSD, *n* = 4 biological repeats, 7–12 cells per repeat. (E) Orthogonal views of ACM-treated neurons following timelapse acquisition (>60 min). (F) Representative image of neurons following 24 h incubation with ACM and immunolabeling against GFP with an inset showing orthogonal views. *∗∗p* < 0.01; ns, not significant. See also Video S2.

We hypothesized that MitoTracker could also be transferred via ACM. To explore this, ACM (generated by incubation of neuronal media with dual labeled astrocytes for 24 h) was applied to neurons during image acquisition (Figure 3A). Notably, the MitoTracker fluorescence significantly increased in neurons throughout the image acquisition period, while the GFP-OMM signal did not increase above background (Figures 3B–3D andS3A; Video S2). Post-timelapse imaging confirmed that MitoTracker, but not GFP-OMM, was located within neurons (Figure 3B). However, because we did not perform extracellular fluorescence quenching and/or live washing controls during imaging, we cannot fully exclude the possibility that a fraction of the MitoTracker signal reflects surface-associated dye rather than strictly intracellular signal. To determine whether MitoTracker that transferred via ACM incubation was due to dye leak into the media, we depleted mitochondria/EVs by filtration^15^ or further centrifugation of ACM.^2^ Both filtration and centrifugation reduced but did not eliminate the MitoTracker fluorescence intensity in neurons, reflecting a reduction in mitochondrial dye transfer (Figures 3B and 3C). This suggests that cell components that are depleted by filtration or centrifugation can augment transfer of mitochondrial dyes. This was confirmed by comparison of the resuspended mitochondria/EV pellet and the supernatant, which both showed similarly reduced levels of MitoTracker transfer to neurons relative to ACM (Figures S3D and S3E). Therefore, the MitoTracker transfer observed is caused by a combination of both dye released into the media as well as the presence of cell components that can augment this transfer. After 24 h, we were still unable to detect GFP-OMM uptake into neurons whereas MitoTracker was still present within neurons (Figure 3F). Therefore, our findings suggest that MitoTracker can transfer via ACM independently of mitochondrial transfer.

Video S2. MitoTracker transfers from ACM to neurons without transfer of mitochondria, related to Figure 3 Neurons expressing mTagBFP2 (blue) are incubated with ACM from astrocytes expressing GFP-OMM (green) and labeled with MitoTracker dye (magenta).

## Discussion

In summary, we have shown that the mitochondrial dye MitoTracker rapidly transfers from both astrocytes and ACM to neurons, even when there is no evidence of organelle transfer using a robust, genetically encoded label. Therefore, MitoTracker transfer does not strictly correspond to mitochondrial transfer and in our opinion should not be used to investigate IMT.

We were unable to identify transfer of astrocytic mitochondria to neurons using genetically encoded fluorophores. We recognize that light microscopy has much lower throughput than flow cytometry, which is more commonly used to assess transfer. However, the methods used here provide the subcellular resolution to distinguish whether mitochondria are internalized or adhered to the outside of neurons. Furthermore, it remains possible that neuronal stress such as ischemia could increase IMT; however, this needs to be confirmed using genetically encoded mitochondrial labels. As such, we do not refute that mitochondrial transfer from astrocytes to neurons can occur. This is supported by the existing evidence for mitochondrial transfer in studies using genetically encoded mitochondrial labels.^1^^,^^2^^,^^3^^,^^23^ Our data do, however, open the possibility that IMT from astrocytes to neurons is less common than previously reported.

While we do not identify the mechanism of MitoTracker dye transfer in this study, we do show that MitoTracker can transfer from both the mitochondria/EV pellet and the mitochondria/EV-depleted media. One possible route of MitoTracker release from astrocytes is through the ATP-dependent efflux pump P-glycoprotein, for which MitoTracker is a known substrate.^24^^,^^25^

The release of free or EV-mitochondria by astrocytes is now well established through electron microscopy, western blots, and ATP assays of conditioned media.^2^^,^^14^^,^^15^ In support of this, we were able to identify extracellular astrocytic mitochondria, most of which were in close proximity to, but not internalized by, adjacent neurons. While the work described here brings into question IMT to neurons, we do not deny the potential benefits of mitochondrial release by astrocytes. For example, there is evidence suggesting that ACM or mitochondrial transplantation can be neuroprotective, both *in vitro* and *in vivo*, against ischemic insults.^2^^,^^11^^,^^12^^,^^15^ However, we suggest a reconsideration of whether mitochondrial transfer is itself neuroprotective, or if the presence of mitochondria and/or EVs in the extracellular space is sufficient. Alternatively, the neuroprotective effect of ACM could be elicited by EVs not containing mitochondria.

In this study we used cell-type-specific expression of genetically encoded fluorophores to allow us to determine the cell origin of observed mitochondria. Going forward, the optimal tools for studying IMT would enable specific detection of mitochondria that have undergone transfer. Existing examples include split-GFP and split-luciferase assays, where complementary parts of the reporter are expressed in donor and acceptor mitochondria, and upon reconstitution can identify integration of transferred mitochondria into the existing mitochondrial network.^26^^,^^27^ Alternatively, a new method—MitoTracer—can enable clear, binary identification of cells with and without transferred mitochondria as mitochondrial transfer causes acceptor cells to switch expression from DsRed-Express2 to eGFP.^28^

In summary, in our opinion MitoTracker dye should not be used to study IMT.^18^^,^^21^ Moreover, we suggest that previous findings are re-examined in the context of dye transfer. Further experiments using appropriate genetically encoded mitochondrial labels will be necessary to validate and improve the understanding of mitochondrial transfer from astrocytes to neurons.

### Limitations of the study

The light microscopy techniques used in this study are low throughput compared to flow cytometry, which is more commonly used to assess IMT. Furthermore, these experiments were conducted under physiological conditions rather than inducing neuronal stress which has been suggested to upregulate IMT. This could explain why genuine IMT was not detected in this study. It is possible that if genuine IMT was to occur, the signal intensity of transferred mitochondria would be bright enough to distinguish between donor mitochondria and dye that has transferred. Investigating this possibility will require future experimentation combining MitoTracker with independent mitochondrial labels in different biological contexts.

## Resource availability

### Lead contact

Requests for additional information, resources, or reagents should be directed to the lead contact, Michael J. Devine (michael.devine@crick.ac.uk) who will provide the requested materials and address any inquiries.

### Materials availability

Plasmids generated in this study are available from the lead contact upon request.

### Data and code availability

• Microscopy data are available from the lead contact upon request.
• The macro used for imaging analysis is available in this paper’s supplemental information (methods S2).
• Any additional information required to reanalyze the data reported in this paper is available from the lead contact upon request.

## Acknowledgments

The authors thank the Devine laboratory for their input, particularly Jonathan Spencer for proofreading and Yulia Sudarikova for her assistance with macros for image analysis, respectively. We thank Michael Way for his feedback on the manuscript. We thank the following core facilities at the Francis Crick Institute: Advanced Light Microscopy for imaging support, the Biological Research Facility for assistance with animal work, and Vector Core for plasmid and virus preparation. This work was supported by 10.13039/100010438The Francis Crick Institute, which receives its core funding from 10.13039/501100000289Cancer Research UK (CC2206), the 10.13039/501100000265UK Medical Research Council (CC2206), and the 10.13039/100010269Wellcome Trust (CC2206). K.L.H. was supported by funding from MSD and the MRC as part of the Crick-MSD Research Alliance. Schematics and diagrams were created in BioRender. Hole, K. (2025) https://BioRender.com/olqio7s, https://BioRender.com/vas9we6, https://BioRender.com/c00spf5.

## Author contributions

K.L.H., M.J.D., N.J.C., J.H.H., and J.B. conceived and designed the study; K.L.H. performed all the experiments and analysis; E.M. established the transgenic mouse lines; M.S. designed the plasmids and generated the viruses; R.N. assisted with experimental design and preparation of primary neurons; P.C. assisted with preparation of primary astrocytes; K.L.H. and M.J.D. wrote the original manuscript, and all authors contributed to improving it.

## Declaration of interests

The authors declare no competing interests.

## STAR★Methods

### Key resources table

**Table:** 

REAGENT or RESOURCE	SOURCE	IDENTIFIER
**Antibodies**		

Rabbit Polyclonal anti-GFP	Abcam	Cat # ab6556; RRID:AB_305564
Chicken anti-Rabbit IgG (H + L) Cross-Adsorbed Secondary Antibody, Alexa Fluor™ 488	Invitrogen	Cat #A-21441; RRID:AB_2535859

**Bacterial and virus strains**		

pAAV-hSyn-mTagBFP2	This study	N/A
pAAV-hSyn-mito-mTagBFP2	This study	N/A
Lenti-GfaABC1D-mito-DsRed2	This study	N/A

**Chemicals, peptides, and recombinant proteins**		

CellTracker™ Deep Red	Invitrogen	Cat #C34565
CellTracker™ Orange CMTMR	Invitrogen	Cat #C2927
MitoTracker™ Red CMXROS	Invitrogen	Cat #M7512
Image-iT™ TMRM Reagent	Invitrogen	Cat #I34361
MitoTracker™ Deep Red FM	Invitrogen	Cat #M22426
MitoTracker™ Green FM	Invitrogen	Cat #M7514
Poly-L-Lysine hydrobromide	Sigma Aldrich	Cat #P2636
Borate Buffer 0.1M, pH 8.5	bioWORLD	Cat #40121000
Horse Serum, heat inactivated, New Zealand origin	Gibco	Cat #26050088
Sodium Pyruvate 100mM	Gibco	Cat #11360070
45% Glucose Solution	Corning	Cat #25-037-CI
MEM	Gibco	Cat #31095029
HBSS (10×), no Ca^2+^, no Mg^2+^, phenol red	Gibco	Cat #14180046
HEPES (1M)	Gibco	Cat #15630080
Trypsin-EDTA (0.05%), phenol red	Gibco	Cat #25300054
DNase I	Roche	Cat #11284932001
B27 Supplement	Gibco	Cat #17504044
Neurobasal Media	Gibco	Cat #21103049
GlutaMAX Supplement	Gibco	Cat #35050038
BrainPhys Neuronal Medium	STEMCELL Technologies	Cat #05790
NeuroCult SM1 Neuronal Supplement	STEMCELL Technologies	Cat #05711
DPBS, no Ca^2+^, no Mg^2+^	Gibco	Cat #14190144
Papain	Worthington Biochemicals	Cat #LS003124
Hibernate-A Medium	Gibco	Cat #A1247501
DMEM, high glucose, pyruvate	Gibco	Cat #41966029
Fetal Bovine Serum, Value	Gibco	Cat #A52567
Recovery Cell Culture Freezing Medium	Gibco	Cat #11560446
16% Paraformaldehyde	Electron Microscopy Sciences	Cat #1570
Sucrose	Sigma Aldrich	Cat #S0389
Bovine Serum Albumin	Sigma Aldrich	Cat #A7906
ProLong Glass Antifade Mountant	Invitrogen	Cat #P36980
DAPI	Sigma Aldrich	Cat #D9542

**Experimental models: Cell lines**		

Primary murine cortical astrocytes derived from GFAP-cre x MitoTag mice, postnatal day 0–2	Generated by the authors of this paper	N/A
Primary murine cortical astrocytes derived from C57BL/6J mice, postnatal day 0–2	Generated by the authors of this paper	N/A
Primary cortical neurons derived from C57BL/6J mice, embryonic day 16.5	Generated by the authors of this paper	N/A

**Experimental models: Organisms/strains**		

Mouse: C57BL/6J	The Jackson Laboratory	000664; RRID:IMSR_JAX:000664
Mouse: B6.Cg-Tg(Gfap-cre)77.6Mvs/2J	The Jackson Laboratory	024098; RRID:IMSR_JAX:024098
Mouse: B6N.Cg-Gt(ROSA)26Sor ^tm1(CAG−EGFP∗)Thm^/J	The Jackson Laboratory	032675; RRID:IMSR_JAX:032675

**Recombinant DNA**		

pAAV2/1	James M. Wilson	Addgene plasmid #112862; RRID:Addgene_112862
pDsRed-Mito-7	Michael Davidson	Addgene plasmid #55838; RRID:Addgene_55838
pCAG mito-mTagBFP2	Franck Polleux	Addgene plasmid #105011; RRID:Addgene_105011
pZac2.1-GfaABC1D-TurboID(full)-HA-GPI	Scott Soderling	Addgene plasmid # 166055; RRID:Addgene_166055

**Software and algorithms**		

Fiji (2.16.0)	Schindelin et al.^29^	https://imagej.net/software/fiji/
Microvolution®	Microvolution	https://www.microvolution.com/
Prism 10.4.1.	GraphPad	https://www.graphpad.com/
Micro-Manager v2.1.0	Edelstein et al.^30^	https://micro-manager.org/

**Other**		

Mr Frosty Freezing Container	Thermo Scientific	Cat #5100-0001
Attofluor Cell Chamber, for microscopy	Invitrogen	Cat #A7816
Confocal microscope	Visitech International	VT-iSIM

### Experimental model and study participant details

#### Animals

Animal work was approved by the Francis Crick ethical committee and performed under UK home office licence PP3668665. All animal procedures were carried out at the Francis Crick Institute in accordance with the regulatory standards of the UK Home Office (ASPA 1986 including Amendment Regulations 2012). Mice were housed and bred under specific pathogen-free conditions (SPF) in individually ventilated cages under a 12h light–dark cycle at ambient temperature (19°C–21°C) and humidity (45–55%). Standard food and water were provided *ad libitum*. Additional information can be found in Table S1. Neurons were derived from E16.5 embryos of C57BL/6J RRID:IMSR_JAX:000664, mice of either sex. B6.Cg-Tg(Gfap-cre)77.6Mvs/2J - RRID:IMSR_JAX:024098 (GFAP-Cre) mice and B6N.Cg-Gt(ROSA)26Sortm1(CAG-EGFP∗)Thm/J - RRID:IMSR_JAX:032675 (MitoTag) mice were separately rederived to C57BL/6J mice and backcrossed for 2 and 8 generations respectively, before crossing to generate GFAP-cre x MitoTag mice. The breeding strategy was optimised to account for known potential occurrence of germline deletion of the floxed allele when breeding from males as previously reported^31^ – only female GFAP-cre +ve mice were used for breeding. Astrocytes were generated from P0-2 MitoTag x GFAP-cre pups of either sex.

#### Cell culture

##### Primary neuron culture

Primary cortical neurons were prepared as previously described.^32^ All materials are from Gibco unless stated otherwise. Poly-L-Lysine (PLL, Sigma Aldrich) was prepared in borate buffer (0.1M, pH 8.5) to a stock concentration of 2mg/mL, filter sterilised and stored in aliquots at −20°C until use. The day before culture, glass coverslips (GG-25-1.5H-Pre, Neuvitro) were pre-coated overnight with 0.5mg/mL PLL (diluted in dH_2_O) at 37°C. The following day, coverslips were washed twice in dH2O and left in attachment media (10% heat inactivated horse serum, 1mM sodium pyruvate, 33mM Glucose in MEM) at 37°C, 5% CO2 until use.

E16.5 embryos were harvested and tissue was kept in ice-cold HBSS (1× HBSS, 10mM HEPES pH7.3, in H_2_O) throughout the dissection. Following decapitation, the brain was removed from the skull and the hemispheres separated. The cortices were dissected and then incubated in 0.05% trypsin containing 10μg/mL DNase I (Sigma) for 8 min at 37°C, 5% CO2 with gentle agitation every 2–3 min. The trypsin was removed and the cortices washed three times in HBSS. Tissue was then triturated 15 times in attachment media containing 10μg/mL DNase I (Sigma) with a P1000 pipette. Dissociated cells were transferred to a new 15mL falcon tube. Cells were counted before plating on the pre-coated coverslips at a density of 125,000 cells per well. 5 h post-plating, media was replaced with maintenance media (2% B27, 1% GlutaMAX, 33mM glucose in Neurobasal). From DIV5, a half media change was undertaken with BrainPhys-Neurocult SM1 (STEMCELL Technologies) every 2–3 days until use at DIV12-14.

##### Primary astrocyte culture

Primary cortical astrocytes were derived from P0-2 homozygous MitoTag x GFAP-cre mice of either sex and isolated as previously described.^33^ Tail tissue was taken for genotyping by Transnetyx. Cortices were incubated in papain solution (20 U/mL papain (Worthington Biochemicals, cat. LS003124) in Hibernate-A) for 15 min at 37°C, 5% CO2, then washed three times PBS-Glucose (0.585% glucose in PBS). The tissue was triturated with a P1000 pipette coated in Fetal Bovine Serum (FBS) in astrocyte media (DMEM, cat. 41966-029 with 10% FBS) with 10μg/mL DNase I (Sigma). Astrocytes were then centrifuged at 750 × g for 5 min and the pellet resuspended in astrocyte media. Cells were plated in uncoated T75 flasks at 1 × 10^6^ cells and incubated at 37°C, 5% CO2. The media was replaced after 24 h and every 2–3 days after until the cells reached confluency. At this point, astrocytes were trypsinised, centrifuged at 300 × g for 10 min and resuspended in recovery cell culture freezing medium (Gibco, 11560446) at 4 million cells per mL. 0.5mL astrocytes were transferred to cryovials and stored in a Mr. Frosty freezing container at −70°C for at least 24 h then transferred to liquid nitrogen for long term storage. When needed, astrocytes were thawed and plated in astrocyte media at 300,000 cells in 60mm dishes. These cells were maintained as before until use.

### Method details

#### Adeno-associated virus infection

AAV-hSyn-mTagBFP2 and AAV-hSyn-mito-mTagBFP2, with AAV2/1 serotype, were created by The Francis Crick Vector Core facility at the Francis Crick Institute. Sequences can be found in Methods S1. pAAV2/1 was a gift from James M. Wilson (Addgene plasmid #112862; http://n2t.net/addgene:112862; RRID:Addgene_112862). pCAG mito-mTagBFP2 was a gift from Franck Polleux (Addgene plasmid #105011; http://n2t.net/addgene:105011; RRID:Addgene_105011).^34^

Neurons were infected at DIV6/7 at a multiplicity of infection (MOI) of 30,000 and 20,000 viral genomes/cell for mTagBFP2 and mito-mTagBFP2 respectively, and allowed to express for 6–7 days prior to experiments.

#### Lentiviral infection

Lenti-GfaABC1D-mito-DsRed2, with VSV-G envelope proteins, was created by The Francis Crick Vector Core facility at the Francis Crick Institute. Sequences can be found in Methods S1. pDsRed2-Mito-7 was a gift from Michael Davidson (Addgene plasmid #55838; http://n2t.net/addgene:55838; RRID:Addgene_55838). pZac2.1-GfaABC1D-TurboID(full)-HA-GPI was a gift from Scott Soderling (Addgene plasmid #166055; http://n2t.net/addgene:166055; RRID:Addgene_166055).^35^

Astrocytes were infected 1 day after thawing and plating at an MOI of 30 viral genomes/cell. The media was changed after 24 h and astrocytes were allowed to express for at least 6 days prior to experiments.

#### Primary astrocyte-neuron co-cultures

Astrocytes for co-culture were labeled with 100nM MitoTracker Red CMXRos (Invitrogen, cat. M7512) and CellTracker Deep Red (1:1000 dilution, CTDR, Invitrogen, cat. C34565) in unsupplemented DMEM for 30 min. Following a single wash in astrocyte media, astrocytes were washed five times with PBS to ensure removal of extracellular dye. Astrocytes were then immediately trypsinized and the cell suspension centrifuged at 300 × g for 10 min before resuspension in BrainPhys-SM1 media. Astrocytes were added to neurons at a ratio of 1:1, keeping neuronal conditioned media at 50%.

For immunofluorescence experiments, co-cultures were fixed after 48-h. For live imaging experiments, astrocytes were added to neurons in the imaging chamber, positions of interest identified and images acquired within 3 min of application.

#### Astrocyte conditioned media

24h prior to imaging, astrocytes were labeled with MitoTracker CMXRos as described above, including the washing steps. Astrocytes were then cultured in BrainPhys-SM1 for 24 h before the astrocyte conditioned media was collected and centrifuged at 2000 × g for 10 min to eliminate any cells or debris present in the media, with the supernatant kept as ACM. For mitochondrial depletion, the ACM was either passed through a 0.22μm filter^15^ or centrifuged at 20,000 × g for 30 min at 4°C to pellet mitochondria and EVs, retaining the supernatant.^2^ For comparison of the mitochondria/EV pellet and the supernatant, the pellet was resuspended in an equivalent volume of BrainPhys-SM1.

For fixed imaging experiments, 1mL neuronal media was replaced with 1mL ACM, and neurons were incubated for 24 h prior to fixing.

For live imaging experiments, neurons were initially imaged in 500μL neuronal conditioned media (t = 0), and then 500μL ACM was applied during acquisition. Images were acquired every 2 min for 62 min. For comparison of supernatant and mitochondria/EV pellet, neurons were incubated with the relevant media for 30 min prior to image acquisition.

#### Immunocytochemistry

Fixing solution (4% paraformaldehyde (Electron Microscopy Sciences, 1570), 4% sucrose in PBS) was prewarmed to 37°C before incubating with cells for 15 min. Following 3 × 5 min washes with PBS, cells were left in PBS at 4°C before use. Cells were permeabilised with 0.1% Triton X-100 in PBS for 5 min, followed by 3 × PBS washes. Cells were blocked for 30 min in blocking buffer (1% BSA (Sigma), 1% horse serum (Gibco) in PBS). Anti-GFP (abcam, ab6556) was diluted in blocking buffer (1:2000). Coverslips were incubated with primary antibodies for 30 min, washed 3 × 5 min with blocking buffer, then incubated with Chicken anti-Rabbit IgG (H + L) Cross-Adsorbed Secondary Antibody, Alexa Fluor 488 (Invitrogen, A-21441, 1:1000) secondary antibody for 30 min. Coverslips were washed for a further 3 × 5 min in blocking buffer before another wash step with for 3 × 5 min in PBS. For DAPI staining, coverslips were incubated with DAPI diluted in PBS (1:1000) for 5 min then washed a further 2 × 5 min in PBS. Coverslips were mounted onto slides using ProLong Glass Antifade Mountant and allowed to cure for 24 h at room temperature before sealing with nail varnish.

#### Microscopy

Confocal imaging was undertaken using a Visitech iSIM microscope on an IX83 microscope body and Micro-Manager v2.1.0 software.^30^ Live imaging was undertaken at 37°C with 5% CO_2_. For live imaging, a 60×, 1.4 NA objective was used. Images were acquired with z-stacks of 15 × 0.5 μm steps for all experiments except the comparison of supernatant and mitochondria/EV pellet, which was undertaken with z-stacks of 30 × 0.25μm steps.

Post-timelapse and fixed imaging was undertaken with a 100×, 1.5 NA objective. Images were acquired with z-stacks of 0.125μm steps. These images were deconvoluted using Microvolution software with 30 iterations and a background of 104.

Widefield microscopy was undertaken with a fluorescent microscope on a Nikon Ti2 microscope body (Evident) with a 20×, 0.75NA objective.

#### Image analysis

All image analysis was undertaken using Fiji (2.16.0).^29^

The percentage of GFP-OMM positive cells in astrocyte cultures was calculated as the number of GFP and DAPI positive cells over the total number of DAPI positive cells. The code used to accomplish this can be found in Methods S2.

CellTracker Deep Red staining of astrocytes was used to identify mitochondria that were “extra-astrocytic”. Each extra-astrocytic mitochondrion identified was then classified as either internalised, adjacent or nonadjacent to neurons based on their proximity to mTagBFP2. Clusters of extra-astrocytic mitochondria that resembled cellular debris were not included in this analysis.

To measure fluorescence intensity over time, a sum-stack z-projection was created. The cell body was outlined using the cell-fill as reference. The mean fluorescence intensity within that area was measured over time for each channel of interest. For df/f0 calculations, f0 relates to the fluorescence before ACM was added. Where necessary, drift correction was undertaken using the Correct 3D Drift plug-in.^36^

For comparison of pellet and supernatant after 30 min of incubation, a sum-stack z-projection was created. To segment the neurons a guassian-blur filter was applied with a sigma (radius) of 2 then the intermodes threshold was applied to create a mask of the mTagBFP2 channel. This mask was then used to measure the MitoTracker fluorescence intensity in neurons. The background was selected from a non-mTagBFP2 positive region and this was subtracted from the neuronal MitoTracker fluorescence intensity to give the final value.

### Quantification and statistical analysis

#### Statistical analysis

Statistics were performed using GraphPad Prism 10.4.1. Details regarding the exact values of *n* can be found in the figure legends. All statistics are undertaken with the *n* relating to the number of biological repeats. All error bars shown are standard deviation.

## Supplemental information

Document S1. Figures S1–S3, Table S1, and Methods S1 and S2

Document S2. Article plus supplemental information
