# Supplementary material for: MitoTracker transfers from astrocytes to neurons independently of mitochondria
Source: Cell Rep Methods. 2026 Mar 13;6(3):101338. doi: 10.1016/j.crmeth.2026.101338 (PMC13030966; doi:10.1016/j.crmeth.2026.101338)
Supplement: Document S1. Figures S1–S3, Table S1, and Methods S1 and S2 [file mmc1.pdf]

Cell Reports Methods, Volume *6*

Supplemental information

MitoTracker transfers from astrocytes to neurons independently of mitochondria

Katriona L. Hole, Rosalind Norkett, Emma Russell, Patrick Cottilli, Molly Strom, Jack H. Howden, Nicola J. Corbett, Janet Brownlees, and Michael J. Devine

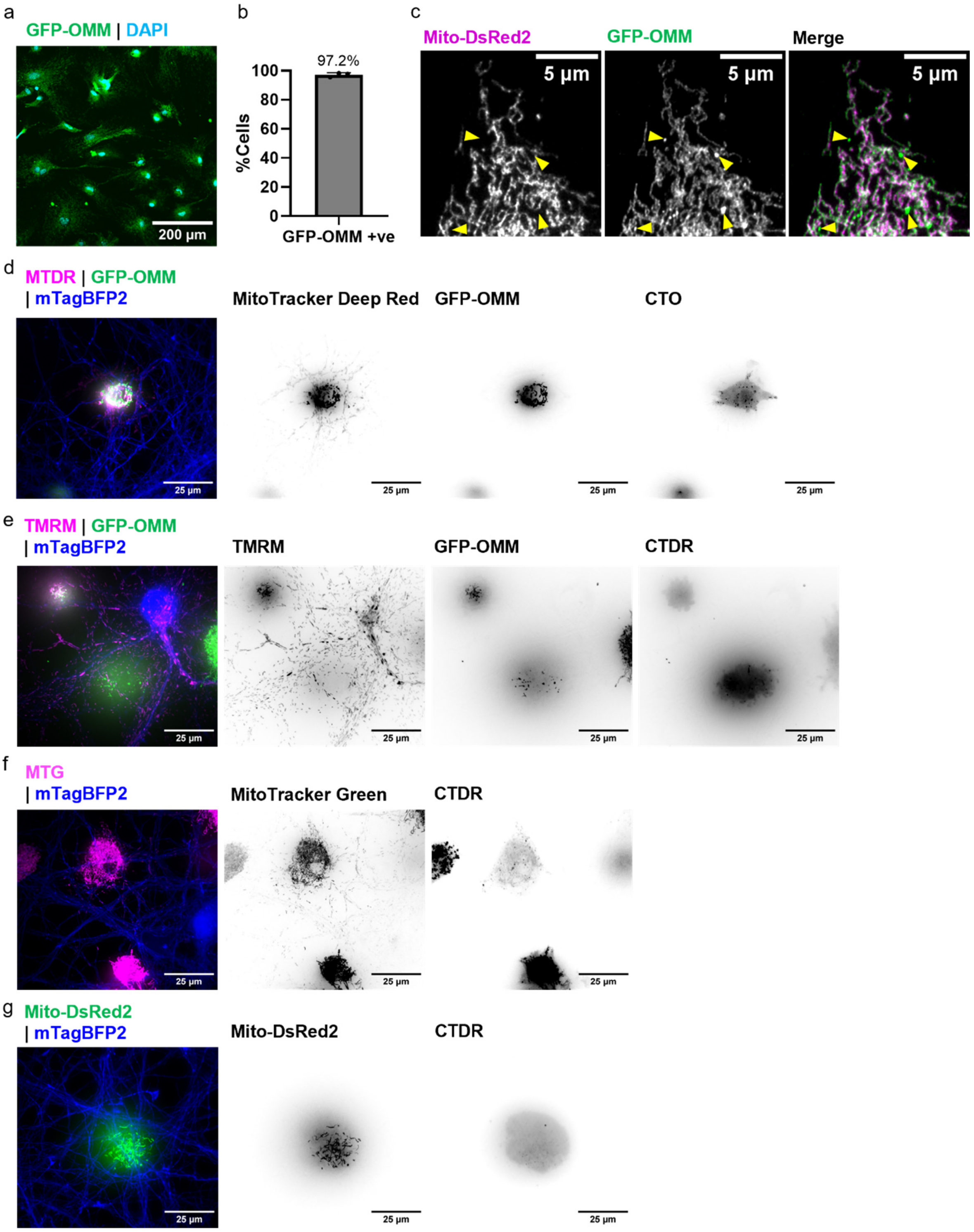

Figure S1. Validation of GFP-OMM labelling and dye transfer with alternative mitochondrial dyes, related to Figure 1. (a) Representative image of primary cortical astrocytes derived from MitoTag x GFAP-cre mice to express GFP-OMM specifically in astrocytes. GFP signal was enhanced with anti-GFP immunofluorescence. (b) Percentage of GFP-OMM +ve cells in primary astrocyte culture. Each repeat relates to separate coverslips of astrocytes each derived from a different pup and cultured at the same time. Data is presented as mean ± SD, with individual biological repeats shown.(c) GFP-OMM expressing astrocyte expressing mito-DsRed2. GFP signal is enhanced with anti-GFP immunofluorescence. GFP-OMM labels all the mitochondria that are also labelled with mito-DsRed2. The yellow arrows highlight GFP-OMM positive, mito-DsRed2 negative mitochondria. (d-g) Astrocytes were labelled with either CellTracker Orange (CTO) or CellTracker Deep Red (CTDR) prior to co-culture with neurons expressing mTagBFP2. Images were acquired after 30 minutes of co-culture. (d,e) GFP-OMM expressing astrocytes were stained with the membrane potential sensitive mitochondrial dyes (d) MitoTracker Deep red (MTDR) or (e) TMRM prior to co-culture with neurons.(f) Astrocytes were stained with the membrane potential insensitive dye MitoTracker Green (MTG) prior to co-culture with neurons. (g) Astrocytes expressing mito-DsRed2 were co-cultured with neurons. All images are maximum projections.

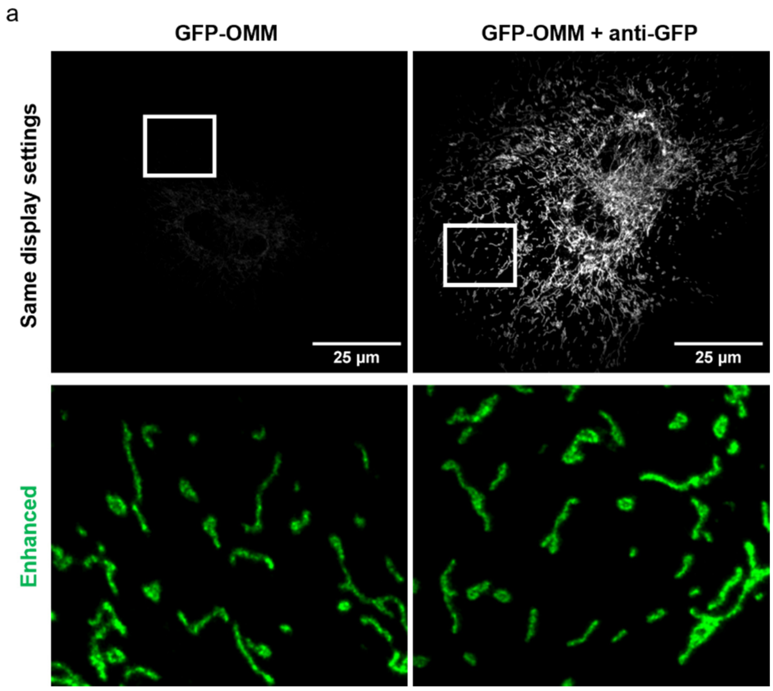

Figure S2. Immunofluorescent labelling of GFP, related to Figure 2. (a) Primary cortical astrocytes expressing GFP-OMM with or without anti-GFP immunofluorescence. The top panel shows a direct comparison with the same display settings, with a clear increase in brightness with the anti-GFP antibody. The bottom panel shows the insets with increased brightness.

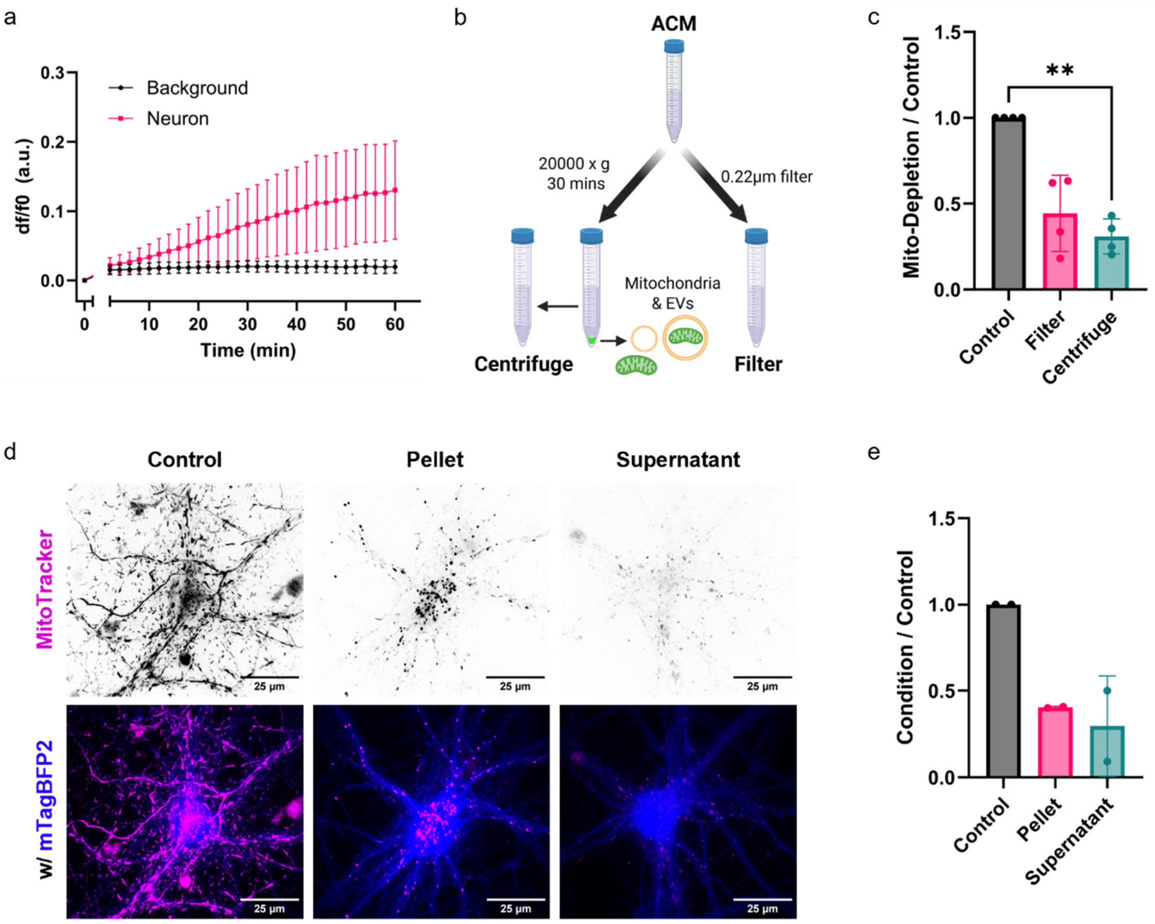

Figure S3. MitoTracker leak into the media does not fully explain MitoTracker transfer from ACM, related to Figure 3. (a) A comparison of the MitoTracker signal within the soma of neurons compared to background after application of ACM over time. (b) A schematic demonstrating the different methods of mitochondrial/EV depletion from ACM. (c) The ratio of somatic fluorescence intensity from mito-depleted ACM (filtered or centrifuged) relative to control ACM (control) after 1 hour incubation. Ratio paired t-test, n = 4 biological repeats, 7-13 cells per repeat. The data is presented as mean ± SD, with individual repeats shown. (d) Example images of neurons after 30 mins of incubation with ACM (control), the resuspended pellet or the supernatant after centrifugation. (e) The ratio of neuronal fluorescence intensity from the pellet, or supernatant relative to control ACM (control) after 30 mins of incubation. n = 2 biological repeats, 5 regions of interest per repeat. The data is presented as mean ± SD, with individual repeats shown. **P < 0.01, ns = not significant.

Table S1. Details of housing and husbandry for mice, related to STAR Methods.

Categories	Details
**Cage/tank/housing system (type and dimensions)**	Individually Ventilated Cages Green line, floor area 500 cm2 (Tecniplast, Italy), Isolators – (PFI Systems, UK)
**Food and water (type, composition, supplier and access)**	2018 Teklad global diet (Envigo, UK) autoclaved before use;<br>Isolators: T.2918CSD irradiated diet (Envigo, UK)<br>Drinking water is mains water passed through RO system and provided in cages by Automated Watering System (Avidity Science, USA)
**Bedding and nesting material**	Bedding: Aspen 4HK (Datesand, UK)<br>Nesting material: Bed R nest (Datesand, UK)
**Temperature and humidity**	Temperature 21oC ± 2oC (in May 2022 temperature in holding rooms was raised to 22 +/- 2)<br>Humidity: 55% RH ± 10%
**Sanitation (frequency of cage/tank water changes, material transferred, water quality)**	Cages are changed as required following SOP on discretionary cage changing with some enrichment and nesting material transferred to new cage to minimise animal stress.<br>Cage cleaning is done via a Tecniplast Pegasus robotic & tunnel washing system and then autoclaved before use. Cage lids and food hoppers are processed via rack washer and then autoclaved as and when required.<br>Avidity automatic watering system is flushed once per day. Animal drinking water is RO treated water with addition of chlorine.
**Social environment (group size and composition/stocking density)**	Mice are group housed with maximum occupancy in accordance with regulatory requirements. Mice with body weight >20g are housed with up to 5 mice per cage. Breeding set ups are pairs and trios. Experimental breeding pairs (to produce pups for primary astrocytes) were separated and the mother housed singly once plugged. Mice are only individually housed singly if required by experiment, for welfare reasons, or if no alternative was available.
**Biosecurity (level)**	A hybrid health monitoring programme which utilizes dirty bedding sentinels and environmental swabs is employed.<br>For mice, at any one time two sentinels are in place per group of racks and are taken for examination when they have been exposed to the environment for approximately six months. Environmental swabs are taken from surfaces exposed to exhaust air from the racks every 4 months.<br>Blood is drawn from sentinels, once only, for serology during the monitoring period. At the end of the exposure period the sentinels are killed by an i.p. overdose of pentobarbitone and subjected to a full necropsy. The pelt is examined for ectoparasites and samples taken include blood, throat and caecal swabs, and faecal pellets. Other swabs and tissues are taken are when deemed necessary. Wet mounts of gut contents are examined for endoparasites and tape tests performed for Syphacia. Gross abnormalities and lesions are recorded and investigated further as necessary.<br>Bacteriology, serology and basic parasitology is carried out by the Crick BRF microbiology laboratory. Some other analyses are outsourced. The choice of agents screened and screening frequencies conform broadly to FELASA recommendations, with some amendments as deemed appropriate by the Veterinary and Animal Health Services Team
**Lighting (type, schedule and intensity)**	12 hrs day /night cycles with 70% light intensity with 15 minutes gradual increase/ decrease in light intensity
**Environmental enrichment**	Cage balconies (Tecniplast, UK), and cardboard mouse houses (Datesand, UK) were provided.
**Sex of the animals**	Pups and embryos of either sex were used.

Methods S1. Details of plasmid sequences, related to STAR Methods.

pAAV hSyn mito-mTagBFP2

ITRs=green

hSynapsin promoter =Red

COX8=pink

mTagBFP2=blue

WPRE=orange

hGH=purple

acatgtcctgcaggcagctgcgcgctcgctcgctcactgaggccgcccgggcgtcgggcgacctttggtcgcccggcctcagtgagcg
agcgagcgcgcagagagggagtggccaactccatcactaggggttcctgcggccgcacgcgtgtgtctagactgcagagggccctg
cgtatgagtgcaagtgggttttaggaccaggatgaggcggggtgggggtgcctacctgacgaccgaccccgacccactggacaagc
acccaacccccattccccaaattgcgcatcccctatcagagagggggaggggaaacaggatgcggcgaggcgcgtgcgcactgcca
gcttcagcaccgcggacagtgccttcgcccccgcctggcggcgcgcgccaccgccgcctcagcactgaaggcgcgctgacgtcactc
gccggtcccccgcaaactccccttcccggccaccttggtcgcgtccgcgccgccgccggcccagccggaccgcaccacgcgaggcgc
gagatagggggcacgggcgcgaccatctgcgctgcggcgccggcgactcagcgctgcctcagtctgcggtgggcagcggaggag
tcgtgtcgtgcctgagagcgcagtcgagaaggtaccggatcctctagagtcgacgccaccatgtccgtcctgacgccgctgctgctgc
ggggcttgacaggctcggcccggcggctcccagtgccgcgcgccaagatccattcgttgggggatccaccggtatgagcgagctgat
taaggagaacatgcacatgaagctgtacatggagggcaccgtggacaaccatcacttcaagtgcacatccgagggcgaaggcaag
ccctacgagggcacccagaccatgagaatcaaggtggtcgagggcggccctctccccttcgccttcgacatcctggctactagcttcct
ctacggcagcaagaccttcatcaaccacacccagggcatccccgacttcttcaagcagtccttccctgagggcttcacatgggagaga
gtcaccacatacgaagacgggggcgtgctgaccgctacccaggacaccagcctccaggacggctgcctcatctacaacgtcaagat
cagaggggtgaacttcacatccaacggccctgtgatgcagaagaaaacactcggctgggaggccttcaccgagacgctgtaccccg
ctgacggcggcctggaaggcagaaacgacatggccctgaagctcgtgggcgggagccatctgatcgcaaacgccaagaccacata
tagatccaagaaacccgctaagaacctcaagatgcctggcgtctactatgtggactacagactggaaagaatcaaggaggccaaca
acgagacctacgtcgagcagcacgaggtggcagtggccagatactgcgacctccctagcaaactggggcacaagcttaattaagaa
ttcgatatcaagcttatcgataatcaacctctggattacaaaatttgtgaaagattgactggtattcttaactatgttgctccttttacgcta
tgtggatacgctgctttaatgcctttgtatcatgctattgcttcccgtatggctttcattttctcctccttgtataaatcctggttgctgtctctt
tatgaggagttgtggcccgttgtcaggcaacgtggcgtggtgtgcactgtgtttgctgacgcaacccccactggttggggcattgccac
cacctgtcagctcctttccgggactttcgctttccccctccctattgccacggcggaactcatcgccgcctgccttgcccgctgctggaca
ggggctcggctgttgggcactgacaattccgtggtgttgtcggggaaatcatcgtcctttccttggctgctcgcctatgttgccacctgg
attctgcgcgggacgtccttctgctacgtcccttcggccctcaatccagcggaccttccttcccgcggcctgctgccggctctgcggcctc
ttccgcgtcttcgccttcgccctcagacgagtcggatctccctttgggccgcctccccgcatcgataccgagcgctgctcgagagatcta
cgggtggcatccctgtgacccctccccagtgcctctcctggccctggaagttgccactccagtgcccaccagccttgtcctaataaaatt
aagttgcatcattttgtctgactaggtgtccttctataatattatggggtggaggggggtggtatggagcaaggggcaagttgggaaga
caacctgtagggcctgcggggtctattgggaaccaagctggagtgcagtggcacaatcttggctcactgcaatctccgcctcctgggt
tcaagcgattctcctgcctcagcctcccgagttgttgggattccaggcatgcatgaccaggctcagctaattttgtttttttggtagagac
ggggtttcaccatattggccaggctggtctccaactcctaatctcaggtgatctacccaccttggcctcccaaattgctgggattacagg
cgtgaaccactgctcccttccctgtccttctgattttgtaggtaaccacgtgcggaccgagcggccgcaggaacccctagtgatggagt
tggccactccctctctgcgcgctcgctcgctcactgaggccgggcgaccaaaggtcgcccgacgcccgggctttgcccgggcggcctc
agtgagcgagcgagcgcgcagctgcctgcaggggcgcctgatgcggtattttctccttacgcatctgtgcggtatttcacaccgcatac
gtcaaagcaaccatagtacgcgccctgtagcggcgcattaagcgcggcgggtgtggtggttacgcgcagcgtgaccgctacacttgc
cagcgccttagcgcccgctcctttcgctttcttcccttcctttctcgccacgttcgccggctttccccgtcaagctctaaatcgggggctcc
ctttagggttccgatttagtgctttacggcacctcgaccccaaaaaacttgatttgggtgatggttcacgtagtgggccatcgccctgat

agacggtttttcgccctttgacgttggagtccacgttctttaatagtggactcttgttccaaactggaacaacactcaactctatctcggg
ctattcttttgatttataagggattttgccgatttcggtctattggttaaaaaatgagctgatttaacaaaaatttaacgcgaattttaaca
aaatattaacgtttacaattttatggtgcactctcagtacaatctgctctgatgccgcatagttaagccagccccgacacccgccaacac
ccgctgacgcgccctgacgggcttgtctgctcccggcatccgcttacagacaagctgtgaccgtctccgggagctgcatgtgtcagag
gttttcaccgtcatcaccgaaacgcgcgagacgaaagggcctcgtgatacgcctatttttataggttaatgtcatgataataatggtttct
tagacgtcaggtggcacttttcggggaaatgtgcgcggaacccctatttgtttatttttctaaatacattcaaatatgtatccgctcatga
gacaataaccctgataaatgcttcaataatattgaaaaaggaagagtatgagtattcaacatttccgtgtcgcccttattcccttttttgc
ggcattttgccttcctgtttttgctcacccagaaacgctggtgaaagtaaaagatgctgaagatcagttgggtgcacgagtgggttacat
cgaactggatctcaacagcggtaagatccttgagagttttcgccccgaagaacgttttccaatgatgagcacttttaaagttctgctatg
tggcgcggtattatcccgtattgacgccgggcaagagcaactcggtcgccgcatacactattctcagaatgacttggttgagtactcac
cagtcacagaaaagcatcttacggatggcatgacagtaagagaattatgcagtgctgccataaccatgagtgataacactgcggcca
acttacttctgacaacgatcggaggaccgaaggagctaaccgcttttttgcacaacatgggggatcatgtaactcgccttgatcgttgg
gaaccggagctgaatgaagccataccaaacgacgagcgtgacaccacgatgcctgtagcaatggcaacaacgttgcgcaaactatt
aactggcgaactacttactctagcttcccggcaacaattaatagactggatggaggcggataaagttgcaggaccacttctgcgctcg
gcccttccggctggctggtttattgctgataaatctggagccggtgagcgtgggtctcgcggtatcattgcagcactggggccagatgg
taagccctcccgtatcgtagttatctacacgacggggagtcaggcaactatggatgaacgaaatagacagatcgctgagataggtgc
ctcactgattaagcattggtaactgtcagaccaagtttactcatatatactttagattgatttaaaacttcatttttaatttaaaaggatcta
ggtgaagatcctttttgataatctcatgaccaaaatcccttaacgtgagttttcgttccactgagcgtcagaccccgtagaaaagatcaa
aggatcttcttgagatcctttttttctgcgcgtaatctgctgcttgcaaacaaaaaaaccaccgctaccagcggtggtttgtttgccggat
caagagctaccaactctttttccgaaggtaactggcttcagcagagcgcagataccaaatactgttcttctagtgtagccgtagttaggc
caccacttcaagaactctgtagcaccgcctacatacctcgctctgctaatcctgttaccagtggctgctgccagtggcgataagtcgtgt
cttaccgggttggactcaagacgatagttaccggataaggcgcagcggtcgggctgaacggggggttcgtgcacacagcccagcttg
gagcgaacgacctacaccgaactgagatacctacagcgtgagctatgagaaagcgccacgcttcccgaagggagaaaggcggaca
ggtatccggtaagcggcagggtcggaacaggagagcgcacgagggagcttccagggggaaacgcctggtatctttatagtcctgtc
gggtttcgccacctctgacttgagcgtcgatttttgtgatgctcgtcaggggggcggagcctatggaaaaacgccagcaacgcggcctt
tttacggttcctggccttttgctggccttttgctc

pAAV hSyn mTagBFP2

ITRs=green

hSynapsin promoter =Red

mTagBFP2=blue

WPRE=orange

hGH=purple

acatgtcctgcaggcagctgcgcgctcgctcgctcactgaggccgcccgggcgtcgggcgacctttggtcgcccggcctcagtgagcg
agcgagcgcgcagagagggagtggccaactccatcactaggggttcctgcggccgcacgcgtgtgtctagactgcagagggccctg
cgtatgagtgcaagtgggttttaggaccaggatgaggcggggtgggggtgcctacctgacgaccgaccccgacccactggacaagc
acccaacccccattccccaaattgcgcatcccctatcagagagggggaggggaaacaggatgcggcgaggcgcgtgcgcactgcca
gcttcagcaccgcggacagtgccttcgcccccgcctggcggcgcgcgccaccgccgcctcagcactgaaggcgcgctgacgtcactc
gccggtcccccgcaaactccccttcccggccaccttggtcgcgtccgcgccgccgccggcccagccggaccgcaccacgcgaggcgc
gagataggggggcacgggcgcgaccatctgcgctgcggcgccggcgactcagcgctgcctcagtctgcggtgggcagcggaggag
tcgtgtcgtgcctgagagcgcagtcgagaaggtaccgccaccatgagcgagctgattaaggagaacatgcacatgaagctgtacatg
gagggcaccgtggacaaccatcacttcaagtgcacatccgagggcgaaggcaagccctacgagggcacccagaccatgagaatca
aggtggtcgagggcggccctctccccttcgccttcgacatcctggctactagcttcctctacggcagcaagaccttcatcaaccacacc
cagggcatccccgacttcttcaagcagtccttccctgagggcttcacatgggagagagtcaccacatacgaagacgggggcgtgctg
accgctacccaggacaccagcctccaggacggctgcctcatctacaacgtcaagatcagaggggtgaacttcacatccaacggccct
gtgatgcagaagaaaacactcggctgggaggccttcaccgagacgctgtaccccgctgacggcggcctggaaggcagaaacgaca
tggccctgaagctcgtgggcgggagccatctgatcgcaaacgccaagaccacatatagatccaagaaacccgctaagaacctcaag
atgcctggcgtctactatgtggactacagactggaaagaatcaaggaggccaacaacgagacctacgtcgagcagcacgaggtggc
agtggccagatactgcgacctccctagcaaactggggcacaagcttaattaagaattcgatatcaagcttatcgataatcaacctctg
gattacaaaatttgtgaaagattgactggtattcttaactatgttgctccttttacgctatgtggatacgctgctttaatgcctttgtatcat
gctattgcttcccgtatggctttcattttctcctccttgtataaatcctggttgctgtctctttatgaggagttgtggcccgttgtcaggcaac
gtggcgtggtgtgcactgtgtttgctgacgcaacccccactggttggggcattgccaccacctgtcagctcctttccgggactttcgcttt
ccccctccctattgccacggcggaactcatcgccgcctgccttgcccgctgctggacaggggctcggctgttgggcactgacaattccg
tggtgttgtcggggaaatcatcgtcctttccttggctgctcgcctatgttgccacctggattctgcgcgggacgtccttctgctacgtccct
tcggccctcaatccagcggaccttccttcccgcggcctgctgccggctctgcggcctcttccgcgtcttcgccttcgccctcagacgagtc
ggatctccctttgggccgcctccccgcatcgataccgagcgctgctcgagagatctacgggtggcatccctgtgacccctccccagtgc
ctctcctggccctggaagttgccactccagtgcccaccagccttgtcctaataaaattaagttgcatcattttgtctgactaggtgtccttc
tataatattatggggtggaggggggtggtatggagcaaggggcaagttgggaagacaacctgtagggcctgcggggtctattggga
accaagctggagtgcagtggcacaatcttggctcactgcaatctccgcctcctgggttcaagcgattctcctgcctcagcctcccgagtt
gttgggattccaggcatgcatgaccaggctcagctaattttgtttttttggtagagacggggtttcaccatattggccaggctggtctcc
aactcctaatctcaggtgatctacccaccttggcctcccaaattgctgggattacaggcgtgaaccactgctcccttccctgtccttctga
ttttgtaggtaaccacgtgcggaccgagcggccgcaggaacccctagtgatggagttggccactccctctctgcgcgctcgctcgctca
ctgaggccgggcgaccaaaggtcgcccgacgcccgggctttgcccgggcggcctcagtgagcgagcgagcgcgcagctgcctgca
ggggcgcctgatgcggtattttctccttacgcatctgtgcggtatttcacaccgcatacgtcaaagcaaccatagtacgcgccctgtagc
ggcgcattaagcgcggcgggtgtggtggttacgcgcagcgtgaccgctacacttgccagcgccttagcgcccgctcctttcgctttcttc
ccttcctttctcgccacgttcgccggctttccccgtcaagctctaaatcgggggctccctttagggttccgatttagtgctttacggcacct
cgaccccaaaaaacttgatttgggtgatggttcacgtagtgggccatcgccctgatagacggtttttcgccctttgacgttggagtccac
gttctttaatagtggactcttgttccaaactggaacaacactcaactctatctcgggctattcttttgatttataagggattttgccgatttc
ggtctattggttaaaaaatgagctgatttaacaaaaatttaacgcgaattttaacaaaatattaacgtttacaattttatggtgcactctc
agtacaatctgctctgatgccgcatagttaagccagccccgacacccgccaacacccgctgacgcgccctgacgggcttgtctgctcc
cggcatccgcttacagacaagctgtgaccgtctccgggagctgcatgtgtcagaggttttcaccgtcatcaccgaaacgcgcgagacg
aaagggcctcgtgatacgcctatttttataggttaatgtcatgataataatggtttcttagacgtcaggtggcacttttcggggaaatgtg

cgcggaacccctatttgtttatttttctaaatacattcaaatatgtatccgctcatgagacaataaccctgataaatgcttcaataatattg
aaaaaggaagagtatgagtattcaacatttccgtgtcgcccttattcccttttttgcggcattttgccttcctgtttttgctcacccagaaac
gctggtgaaagtaaaagatgctgaagatcagttgggtgcacgagtgggttacatcgaactggatctcaacagcggtaagatccttga
gagttttcgccccgaagaacgttttccaatgatgagcacttttaaagttctgctatgtggcgcggtattatcccgtattgacgccgggcaa
gagcaactcggtcgccgcatacactattctcagaatgacttggttgagtactcaccagtcacagaaaagcatcttacggatggcatga
cagtaagagaattatgcagtgctgccataaccatgagtgataacactgcggccaacttacttctgacaacgatcggaggaccgaagg
agctaaccgcttttttgcacaacatgggggatcatgtaactcgccttgatcgttgggaaccggagctgaatgaagccataccaaacga
cgagcgtgacaccacgatgcctgtagcaatggcaacaacgttgcgcaaactattaactggcgaactacttactctagcttcccggcaa
caattaatagactggatggaggcggataaagttgcaggaccacttctgcgctcggcccttccggctggctggtttattgctgataaatc
tggagccggtgagcgtgggtctcgcggtatcattgcagcactggggccagatggtaagccctcccgtatcgtagttatctacacgacg
gggagtcaggcaactatggatgaacgaaatagacagatcgctgagataggtgcctcactgattaagcattggtaactgtcagaccaa
gtttactcatatatactttagattgatttaaaacttcatttttaatttaaaaggatctaggtgaagatcctttttgataatctcatgaccaaa
atcccttaacgtgagttttcgttccactgagcgtcagaccccgtagaaaagatcaaaggatcttcttgagatcctttttttctgcgcgtaat
ctgctgcttgcaaacaaaaaaaccaccgctaccagcggtggtttgtttgccggatcaagagctaccaactctttttccgaaggtaactg
gcttcagcagagcgcagataccaaatactgttcttctagtgtagccgtagttaggccaccacttcaagaactctgtagcaccgcctaca
tacctcgctctgctaatcctgttaccagtggctgctgccagtggcgataagtcgtgtcttaccgggttggactcaagacgatagttaccg
gataaggcgcagcggtcgggctgaacggggggttcgtgcacacagcccagcttggagcgaacgacctacaccgaactgagatacct
acagcgtgagctatgagaaagcgccacgcttcccgaagggagaaaggcggacaggtatccggtaagcggcagggtcggaacagg
agagcgcacgagggagcttccagggggaaacgcctggtatctttatagtcctgtcgggtttcgccacctctgacttgagcgtcgatttttt
gtgatgctcgtcaggggggcggagcctatggaaaaacgccagcaacgcggcctttttacggttcctggccttttgctggccttttgctc

pLenti GfaABC1D mito dsRED

CMV enhancer
5' LTR (truncated)
HIV-1 ψ
RRE
cPPT/CTS
GfaABC1D promoter
COX8 presequence
DsRed2
WPRENoX
5'LTR (truncated)

acgcgttgacattgattattgactagttattaatagtaatcaattacggggtcattagttcatagcccatatatggagttccgcgttacata
acttacggtaaatggcccgcctggctgaccgcccaacgacccccgcccattgacgtcaataatgacgtatgttcccatagtaacgcca
atagggactttccattgacgtcaatgggtggagtatttacggtaaactgcccacttggcagtacatcaagtgtatcatatgccaagtac
gccccctattgacgtcaatgacggtaaatggcccgcctggcattatgcccagtacatgaccttatgggactttcctacttggcagtacat
ctacgtattagtcatcgctattaccatggtgatgcggttttggcagtacatcaatgggcgtggatagcggtttgactcacggggatttcc
aagtctccaccccattgacgtcaatgggagtttgttttggcaccaaaatcaacgggactttccaaaatgtcgtaacaactccgccccat
tgacgcaaatgggcggtaggcgtgtacggtgggaggtctatataagcagcgcgttttgcctgtactgggtctctctggttagaccagat
ctgagcctgggagctctctggctaactagggaacccactgcttaagcctcaataaagcttgccttgagtgcttcaagtagtgtgtgccc
gtctgttgtgtgactctggtaactagagatccctcagacccttttagtcagtgtggaaaatctctagcagtggcgcccgaacagggactt
gaaagcgaaagggaaaccagaggagctctctcgacgcaggactcggcttgctgaagcgcgcacggcaagaggcgaggggcggcg
actggtgagtacgccaaaaattttgactagcggaggctagaaggagagagatgggtgcgagagcgtcagtattaagcgggggaga
attagatcgcgatgggaaaaaattcggttaaggccagggggaaagaaaaaatataaattaaaacatatagtatgggcaagcaggg
agctagaacgattcgcagttaatcctggcctgttagaaacatcagaaggctgtagacaaatactgggacagctacaaccatcccttca
gacaggatcagaagaacttagatcattatataatacagtagcaaccctctattgtgtgcatcaaaggatagagataaaagacaccaa
ggaagctttagacaagatagaggaagagcaaaacaaaagtaagaccaccgcacagcaagcggccgctgatcttcagacctggagg
aggagatatgagggacaattggagaagtgaattatataaatataaagtagtaaaaattgaaccattaggagtagcacccaccaagg
caaagagaagagtggtgcagagagaaaaaagagcagtgggaataggagctttgttccttgggttcttgggagcagcaggaagcact
atgggcgcagcgtcaatgacgctgacggtacaggccagacaattattgtctggtatagtgcagcagcagaacaatttgctgagggct
attgaggcgcaacagcatctgttgcaactcacagtctggggcatcaagcagctccaggcaagaatcctggctgtggaaagatacct a
aaggatcaacagctcctggggatttggggttgctctggaaaactcatttgcaccactgctgtgccttggaatgctagttggagtaataa
atctctggaacagatttggaatcacacgacctggatggagtgggacagagaaattaacaattacacaagcttaatacactccttaatt
gaagaatcgcaaaaccagcaagaaaagaatgaacaagaattattggaattagataaatgggcaagtttgtggaattggtttaacata
acaaattggctgtggtatataaaattattcataatgatagtaggaggcttggtaggtttaagaatagtttttgctgtactttctatagtga
atagagttaggcagggatattcaccattatcgtttcagacccacctcccaaccccgaggggacccgacaggcccgaaggaatagaag
aagaaggtggagagagagacagagacagatccattcgattagtgaacggatcggcactgcgtgcgccaattctgcagacaaatggc
agtattcatccacaattttaaaagaaaaggggggattggggggtacagtgcaggggaaagaatagtagacataatagcaacagac
atacaaactaaagaattacaaaaacaaattacaaaaattcaaaattttcgggtttattacagggacagcagagatccagtttggttaa
ttaaacatatcctggtgtggagtaggggacgctgctctgacagaggctcgggggcctgagctggctctgtgagctggggaggaggca
gacagccaggccttgtctgcaagcagacctggcagcattgggctggccgcccccagggcctcctcttcatgcccagtgaatgactca
ccttggcacagacacaatgttcggggtgggcacagtgcctgcttcccgccgcaccccagcccccctcaaatgccttccgagaagccca
ttgagcaggggggcttgcattgcaccccagcctgacagcctggcatcttgggataaaagcagcacagccccctaggggctgcccttgct
gtgtggcgccaccggcggtggagaacaaggctctattcagcctgtgcccaggaaaggggatcaggggatgcccaggcatggacagt
gggtggcaggggggggagaggagggctgtctgcttcccagaagtccaaggacacaaatgggtgaggggagagctctccccatagct
gggctgcggcccaaccccacccccctcaggctatgccagggggtgttgccaggggcacccgggcatcgccagtctagcccactccttc
ataaagccctcgcatcccaggagcgagcagagccagagcaggttggagaggagacgcatcacctccgctgctcgcaagctttattgc

ggtagtttatcacagttaaattgctaacgcagtcagtgcttctgacacaacagtctcgaacttaagctgcagaagttggtcgtgaggca
ctgggcaggtaagtatcaaggttacaagacaggtttaaggagaccaatagaaactgggcttgtcgagacagagaagactcttgcgtt
tctgataggcacctattggtcttactgacatccactttgcctttctctccacaggtgtccactcccagttcaattacagctcttaaggctag
agtacttaatacgactcactataggctagcgccaccatgtccgtcctgacgccgctgctgctgcggggcttgacaggctcggcccggc
ggctcccagtgccgcgcgccaagatccattcgttggggatccaccggtcgccaccatggcctcctccgagaacgtcatcaccgagtt
catgcgcttcaaggtgcgcatggagggcaccgtgaacggccacgagttcgagatcgagggcgagggcgagggccgcccctacgag
ggccacaacaccgtgaagctgaaggtgaccaagggcggccccctgcccttcgcctgggacatcctgtcccccagttccagtacggc
tccaaggtgtacgtgaagcaccccgccgacatccccgactacaagaagctgtccttccccgagggcttcaagtgggagcgcgtgatg
aacttcgaggacggcggcgtggcgaccgtgacccaggactcctccctgcaggacggctgcttcatctacaaggtgaagttcatcggc
gtgaacttcccctccgacggccccgtgatgcagaagaagaccatgggctgggaggcctccaccgagcgcctgtaccccgcgacgg
cgtgctgaagggcgagacccacaaggccctgaagctgaaggacggcggccactacctggtggagttcaagtccatctacatggcca
agaagcccgtgcagctgcccggctactactacgtggacgccaagctggacatcacctcccacaacgaggactacaccatcgtggag
cagtacgagcgcaccgagggccgccaccacctgttcctgtaggaattcgatatcaagcttatcggtaatcaacctctggattacaaaa
tttgtgaaagattgactggtattcttaactatgttgctccttttacgctatgtggatacgctgctttaatgcctttgtatcatgctattgcttcc
cgtatggctttcattttctcctccttgtataaatcctggttgctgtctctttatgaggagttgtggcccgttgtcaggcaacgtggcgtggtg
tgcactgtgtttgctgacgcaacccccactggttggggcattgccaccacctgtcagctcctttccgggactttcgctttccccctccctat
tgccacggcggaactcatcgccgcctgccttgcccgctgctggacaggggctcggctgttgggcactgacaattccgtggtgttgtcgg
ggaaatcatcgtcctttccttggctgctcgcctgtgttgccacctggattctgcgcgggacgtccttctgctacgtcccttcggccctcaat
ccagcggaccttccttcccgcggcctgctgccggctctgcggcctcttccgcgtcttcgccttcgccctcagacgagtcggatctcccttt
gggccgcctccccgcatcgataccgtcgacctcgagacctagaaaaacatggagcaatcacaagtagcaatacagcagctaccaat
gctgattgtgcctggctagaagcacaagaggaggaggaggtgggttttccagtcacacctcaggtacctttaagaccaatgacttaca
aggcagctgtagatcttagccactttttaaaagaaaaggggggactggaagggctaattcactcccaacgaagacaagatatccttg
atctgtggatctaccacacacaaggctacttccctgattggcagaactacacaccagggccagggatcagatatccactgacctttgg
atggtgctacaagctagtaccagttgagcaagagaaggtagaagaagccaatgaaggagagaacacccgcttgttacaccctgtga
gcctgcatgggatggatgacccggagagagaagtattagagtggaggtttgacagccgcctagcatttcatcacatggcccgagagc
tgcatccggactgtactgggtctctctggttagaccagatctgagcctgggagctctctggctaactagggaacccactgcttaagcct
caataaagcttgccttgagtgcttcaagtagtgtgtgcccgtctgttgtgtgactctggtaactagagatccctcagacccttttagtcag
tgtggaaaatctctagcagggcccgtttaaacccgctgatcagcctcgactgtgccttctagttgccagccatctgttgtttgcccctccc
ccgtgccttccttgaccctggaaggtgccactcccactgtcctttcctaataaaatgaggaaattgcatcgcattgtctgagtaggtgtc
attctattctggggggtggggtggggcaggacagcaagggggaggattgggaagacaatagcaggcatgctggggatgcggtggg
ctctatggcttctgaggcggaaagaaccagctggggctctagggggtatccccacgcgccctgtagcggcgcattaagcgcggcggg
tgtggtggttacgcgcagcgtgaccgctacacttgccagcgccctagcgcccgctcctttcgctttcttcccttcctttctcgccacgttcg
ccggctttccccgtcaagctctaaatcgggggctccctttagggttccgatttagtgctttacggcacctcgaccccaaaaaacttgatt
agggtgatggttcacgtagtgggccatcgccctgatagacggtttttcgccctttgacgttggagtccacgttctttaatagtggactctt
gttccaaactggaacaacactcaaccctatctcggtctattcttttgatttataagggattttgccgatttcggcctattggttaaaaaatg
agctgatttaacaaaaatttaacgcgaattaattctgtggaatgtgtgtcagttagggtgtggaaagtccccaggctccccagcaggca
gaagtatgcaaagcatgcatctcaattagtcagcaaccaggtgtggaaagtccccaggctccccagcaggcagaagtatgcaaagc
atgcatctcaattagtcagcaaccatagtcccgcccctaactccgcccagttccgcccattctccgccccatggctgactaattttttttat
ttatgcagaggccgaggccgcctctgcctctgagctattccagaagtagtgaggaggcttttttggaggcctaggcttttgcaaaaagc
tcccgggagcttgtatatccatttcggatctgatcagcacgtgttgacaattaatcatcggcatagtatatcggcatagtataatacgac
aaggtgaggaactaaaccatggccaagttgaccagtgccgttccggtgctcaccgcgcgcgacgtcgccggagcggtcgagttctgg
accgaccggctcgggttctcccgggacttcgtggaggacgacttcgccggtgtggtccgggacgacgtgaccctgttcatcagcgcgg
tccaggaccaggtggtgccggacaacaccctggcctgggtgtgggtgcgcggcctggacgagctgtacgccgagtggtcggaggtc
gtgtccacgaacttccgggacgcctccgggccggccatgaccgagatcggcgagcagccgtgggggcgggagttcgccctgcgcga
cccggccggcaactgcgtgcacttcgtggccgaggagcaggactgacacgtgctacgagatttcgattccaccgccgccttctatgaa
aggttgggcttcggaatcgttttccgggacgccggctggatgatcctccagcgcggggatctcatgctggagttcttcgcccaccccaa
cttgtttattgcagcttataatggttacaaataaagcaatagcatcacaaatttcacaaataaagcatttttttcactgcattctagttgtg

gtttgtccaaactcatcaatgtatcttatcatgtctgtataccgtcgacctctagctagagcttggcgtaatcatggtcatagctgtttcct
gtgtgaaattgttatccgctcacaattccacacaacatacgagccggaagcataaagtgtaaagcctggggtgcctaatgagtgagct
aactcacattaattgcgttgcgctcactgcccgctttccagtcgggaaacctgtcgtgccagctgcattaatgaatcggccaacgcgcg
gggagaggcggtttgcgtattgggcgctcttccgcttcctcgctcactgactcgctgcgctcggtcgttcggctgcggcgagcggtatca
gctcactcaaaggcggtaatacggttatccacagaatcaggggataacgcaggaaagaacatgtgagcaaaaggccagcaaaag
gccaggaaccgtaaaaaggccgcgttgctggcgtttttccataggctccgcccccctgacgagcatcacaaaaatcgacgctcaagtc
agaggtggcgaaacccgacaggactataaagataccaggcgtttccccctggaagctccctcgtgcgctctcctgttccgaccctgcc
gcttaccggatacctgtccgcctttctcccttcgggaagcgtggcgctttctcatagctcacgctgtaggtatctcagttcggtgtaggtc
gttcgctccaagctgggctgtgtgcacgaaccccccgttcagcccgaccgctgcgccttatccggtaactatcgtcttgagtccaaccc
ggtaagacacgacttatcgccactggcagcagccactggtaacaggattagcagagcgaggtatgtaggcggtgctacagagttctt
gaagtggtggcctaactacggctacactagaagaacagtatttggtatctgcgctctgctgaagccagttaccttcggaaaaagagtt
ggtagctcttgatccggcaaacaaaccaccgctggtagcggtggtttttttgtttgcaagcagcagattacgcgcagaaaaaaaggat
ctcaagaagatcctttgatcttttctacggggtctgacgctcagtggaacgaaaactcacgttaagggattttggtcatgagattatcaa
aaaggatcttcacctagatccttttaaattaaaaatgaagttttaaatcaatctaaagtatatatgagtaaacttggtctgacagttacc
aatgcttaatcagtgaggcacctatctcagcgatctgtctatttcgttcatccatagttgcctgactccccgtcgtgtagataactacgat
acgggagggcttaccatctggccccagtgctgcaatgataccgcgagacccacgctcaccggctccagatttatcagcaataaacca
gccagccggaagggccgagcgcagaagtggtcctgcaactttatccgcctccatccagtctattaattgttgccgggaagctagagta
agtagttcgccagttaatagtttgcgcaacgttgttgccattgctacaggcatcgtggtgtcacgctcgtcgtttggtatggcttcattcag
ctccggttcccaacgatcaaggcgagttacatgatcccccatgttgtgcaaaaaagcggttagctccttcggtcctccgatcgttgtcag
aagtaagttggccgcagtgttatcactcatggttatggcagcactgcataattctcttactgtcatgccatccgtaagatgcttttctgtga
ctggtgagtactcaaccaagtcattctgagaatagtgtatgcggcgaccgagttgctcttgcccggcgtcaatacgggataataccgc
gccacatagcagaactttaaaagtgctcatcattggaaaacgttcttcggggcgaaaactctcaaggatcttaccgctgttgagatcca
gttcgatgtaacccactcgtgcacccaactgatcttcagcatcttttactttcaccagcgtttctgggtgagcaaaaacaggaaggcaa
aatgccgcaaaaaagggaataagggcgacacggaaatgttgaatactcatactcttcctttttcaatattattgaagcatttatcaggg
ttattgtctcatgagcggatacatatttgaatgtatttagaaaaataaacaaataggggttccgcgcacatttccccgaaaagtgccac
ctgacgtcgacggatcgggagatctcccgatcccctatggtgcactctcagtacaatctgctctgatgccgcatagttaagccagtatct
gctccctgcttgtgtgttggaggtcgctgagtagtgcgcgagcaaaatttaagctacaacaaggcaaggcttgaccgacaattgcatg
aagaatctgcttagggttaggcgttttgcgctgcttcgcgatgtacgggccagatat

Methods S2. Macro to count GFP +ve cells, related to Figure S1 and STAR Methods.

```
#@ File (label = "Input directory", style = "directory") input
//#@ File (label = "Output directory", style = "directory") output
#@ String (label = "File suffix", value = ".tif") suffix

processFolder(input);

// function to scan folders/subfolders/files to find files with correct suffix
function processFolder(input) {
	list = getFileList(input);
	list = Array.sort(list);
	for (i = 0; i < list.length; i++) {
		if(File.isDirectory(input + File.separator + list[i]))
			processFolder(input + File.separator + list[i]);
		if(endsWith(list[i], suffix))
			processFile(input, list[i]);
	}
}

function processFile(input, file) {

	run("Bio-Formats Importer", "open=[" + input + File.separator + file + "] color_mode=Default rois_import=[ROI manager] view=Hyperstack stack_order=XYCZT");

	//split channels
	//MT = MitoTag (GFP-OMM)
	img = getTitle();
	run("Split Channels");
	MT = "C1-" + img;
	DAPI = "C2-" + img;

```

```
//MT
selectImage(MT);
run("Enhance Contrast", "saturated=0.35");
run("Gaussian Blur...", "sigma=7");
setAutoThreshold("Huang dark no-reset");
run("Convert to Mask");
run("Fill Holes");

//DAPI
selectImage(DAPI);
run("Enhance Contrast", "saturated=0.35");
run("Gaussian Blur...", "sigma=2");
setAutoThreshold("Otsu dark no-reset");
run("Convert to Mask");
run("Watershed");

//DAPI Count
run("Analyze Particles...", "size=25-Infinity display");
DAPImean = getResult("Mean", 0);
nDAPI = nResults;
run("Clear Results");

//DAPI + MT count
selectImage(MT);
imageCalculator("AND create", DAPI, MT );
DoublePos = "Result of " + DAPI;
selectImage(DoublePos);
run("Analyze Particles...", "size=25-Infinity display");
DoublePosmean = getResult("Area", 0);
nDoublePos = nResults;
```

```
		//table
		selectWindow("percentMT");
		row = Table.size("percentMT");
		Table.set("Image", row, img);
		Table.set("DAPI", row, nDAPI);
		Table.set("MT", row, nDoublePos);
		Table.update("percentMT");

		run("Clear Results");
		close("*");
	}
```
